# New vaccines: challenges of discovery

**DOI:** 10.1111/1751-7915.12397

**Published:** 2016-08-18

**Authors:** Adel Mahmoud

**Affiliations:** ^1^ Molecular Biology and Public PolicyPrinceton UniversityPrincetonNJ08544USA

## Abstract

Vaccines have been a major component of preventing and controlling infectious diseases. The basis for discovery of what protects is reviewed as well as new attempts in utilizing Reverse Vaccinology, RNA‐RNA methods and proteome analysis are adding significantly to our knowledge. The challenge of how to define protective and defined components of microbes is still hampering efforts to discover new vaccines. Recent excitement about immunotherapy of cancer opens the way to develop vaccines against multiple malignancies.

## Introduction

The time frame for this series is challenging. Vaccine discovery and development are usually time consuming spanning sometimes several decades. The reasons are related to the complex nature of microbial or therapeutic targets and other scientific and technical hurdles. Deployment of vaccines whether within a nation or globally is a multistep process that includes financial, political and health infrastructure needs. The underlying reasons for these challenges are equally complex.

We will focus in this article on summarizing historical experiences in vaccine discovery and development. This will be followed by examining the state of the art in addressing the current challenges in vaccine discovery. In addition, the recent experiences with several of the emerging infections (MERS, Ebola and Zika) add a dimension to these global health problems that taxed academics, governments and industry (Plotkin *et al*., 2015). We will then briefly examine the status of therapeutic and cancer vaccines as we expand on scientific approaches to these disease particularly with the introduction of personalized medicine concepts. We will end with a perspective summary of what can be expected by 2020.

## Vaccine discovery: historical perspective

The fundamental empirical basis for most of the currently available human vaccines is based on the assumption that priming the host with exposure to modified microbes or their products leads to protection against subsequent exposure to the native organism. Several variations of this concept have been successfully used as illustrated in Table 1. The basis of most of these vaccines is attenuation, inactivation or produce a crude extract. Conjugation of polysaccharides to protein back bone was first described in 1929, but the first conjugated vaccine for human use was launched in the mid 1990s. All of these vaccines have been proven effective, safe and had a great impact on public health. However, the current safety, regulatory and industry practices expanded these approaches in vaccine discovery to more defined products that are molecularly characterized (Mahmoud, 2011).

**Table 1:** Scientific basis for vaccine discoveries in the past two centuries

Fundamental concept	Disease	Immunogen	Year
Animal microbes	Smallpox	Cowpox	1796
Attenuation	Rabies	Infected tissue	1881
Killed microbes	Cholera	Killed bacteria	1886
Toxoids	Tetanus	Heat treated	1890
Virus propagation in cell culture	Poliomyelitis	Viral culture in cells	1949

What has been exciting in the past two decades is a new generation of vaccines based on better and more precise use of polysaccharide conjugation, cloning of specific antigens and viral reassortment (Table 2). In spite of those achievements, the number of available molecularly defined vaccines is too limited. In a very strict manner, both Hepatitis B and Human papillomavirus protective antigens immunogenicity and protective properties depend on assembly as virus‐like particles (VLP). It is the self‐assembly as VLP that impart that property. Furthermore, immunogenicity and resulting protection is enhanced by manipulating the VLP to become uniform in size and shape, rigid and to contain mature disulphide bonds, to achieve optimal immunological and protective responses in the host.

**Table 2:** Vaccine discovery: what is new at the turn of the 21st century?

Organism	Technique	Immunogen	Formulation
Hepatitis B	Cloning	Surface antigen	Virus‐like particle
*Streptococcus pneumoniae*	Conjugation	Surface polysaccharide	Polysaccharide conjugated to CRIM
Human papilloma virus	Cloning	Capsid proteins	Virus‐like particle
*Neisseria meningitidis* serotype B	Cloning, reverse vaccinology	Three proteins and outer membrane vesicles	Combination of cloned proteins and OMV

The third of these molecularly defined vaccines (for Mening B) is made of three microbial proteins in addition to outer membrane vesicles (OMV) as will be discussed below.

## Current efforts in vaccine discovery

**Figure 1:**
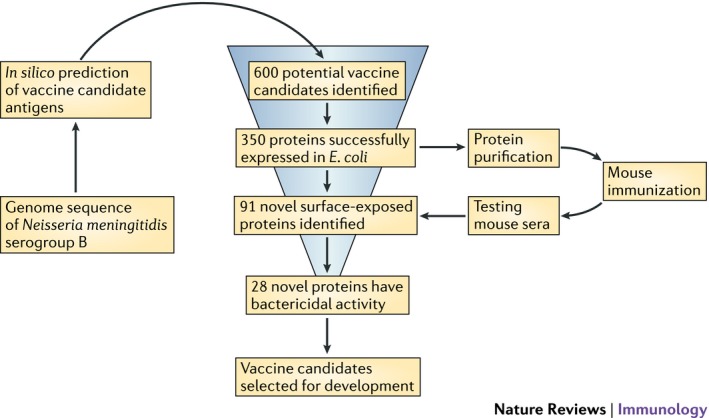
Reverse Vaccinology applied to meningococcus serogroup B. Figure from De Gregorio, E. *et al*. (2014) *Nature Reviews Immunology* **14**:505‐514, with permission.

Cloning and sequencing genomes of several microbial pathogens paved the way to attempt systematic approaches for identification of potential protective microbial components (Table 3). The first meticulous effort was the introduction of Reverse Vaccinology to identify potential protective proteins against *Neisseria meningitidis* serotype B (Fig. 1). Following a laborious and detailed analysis of the proteome of these organisms best described as brutal force, 600 potential proteins were examined. Finally, three were selected to be incorporated in the new vaccine. The basis for this selection starts with identifying all possible proteins encoded by the genome of the microbe that are predicted to be expressed on its surface. The next step involves using immunological tools to identify potential protective molecules which were ultimately included in vaccine preparation. These were Neisserial adhesion A, Heparin Binding Antigen and factor H binding protein. Two other components were added to make an effective vaccine including OMV and aluminium hydroxide (Rappuoli *et al*., 2014). The vaccine was tested in several countries and was originally approved by EMA and then by the FDA to allow its use in outbreak circumstances in the United States (Basta *et al*., 2016). Another monovalent, *N. meningitidis* serotype B vaccine was recently launched as well.

**Table 3:** Newer approaches for characterization of protective antigens

Reverse vaccinology
Antigenome and protectomic technology
Nucleic acid vaccines: DNA and RNA bioinformatics: proteome analysis

One component of the recently developed vaccine for *N. meningitidis* serotype B is the incorporation of OMV or proteins in the final product. These practices were initially used in the 1990s to manufacture a conjugated *Haemophilus influenzae* type B vaccine. Outer membrane vesicles are a component of Gram‐negative bacteria secretion systems. They provide the ability to transport envelope proteins beyond cell surface as well as carrying insoluble proteins and other bacterial molecules all within a confined space. Outer membrane vesicles have been proposed as a platform for vaccine discovery and development particularly as it applies to an overexpression of desired antigens. For example, OMV have been demonstrated as potential protective preparation against several infections in experimental animals and have been used in specific outbreak circumstances, for example, New Zealand meningococcal outbreak. The detailed more recent examination of the structure and components of OMV may lead to their potential wider use as vaccine platform.

## Proteome analysis

With the wide availability of DNA sequencing and protein expression systems several efforts have been in progress to approach the challenge of identifying potential protective antigens from infectious organisms. l Attempt to examine the ‘antigenome’ of pathologic organisms have been initiated but with less clear success. More recently, an attempt was made to reduce the number of molecules that need to be examined to identify potential protective proteins. One such approach was based on proteomics coupled with bioinformatics (Altindis *et al*., 2015). The outcome was a decrease in the total number of proteins to be examined. More recently, one new approach was described based on the assumption that protective bacterial proteins may possess specific signatures. This approach is neither limited to detection of surface expressed molecules nor is it linked to any specific production pathway. This process has been termed ‘Protectome Space’. It has been evaluated for examination of the protectome of *Streptococcus aureus* and Group B Streptococcus. The results not only show confirmation of antigens that were known as protective but also the discovery of others.

The major challenge in identifying potential protective antigens in microbial organism is the lack of tools to identify and characterize these molecules. Immune detection using antibodies or T cells have been used for decades with modest outcome. We know a lot about the details of immune responses and their components but the challenge is how to approach function. The fundamental question is how to differentiate the immune responses intended to detect foreignness from what may be participating in protection of the host. This gap in our knowledge between detection and protection has undermined most of our efforts to use the immune response as a tool for predicting potential protective components of microbes.

## Therapeutic vaccines

The recent success of several approaches to immunotherapy of cancer opened a new chapter in the fight against these diseases. Among the most promising approaches are the use of monoclonal antibodies against cytotoxic T lymphocyte‐associated protein 4 and programmed cell death protein 1 (Lu and Robbins 2016). Great efforts are now being directed at developing vaccines against several oncologic conditions. DNA encoding E6 and E7 of Human papillomavirus administered by electroporation has recently been shown to reverse metaplasia in the cervix in a considerable number of patients (Trimble *et al*., 2015). Similar plans are currently underway to evaluate this approach in other oncologic conditions.

Other approaches to immunotherapy that are being pursued aggressively include adoptive cell therapy and the expanding field of personalized medicine targeting cancer neoantigens (Schumacher and Schreiber, 2015; Sullenger and Nair, 2016). Recognition of these molecules by host T lymphocytes may constitute a significant component of the responses seen following check‐point inhibitor treatments. Furthermore, it may lead to specific treatments directly acting on neoantigens.

## Perspective summation

Vaccines played a central role in controlling many infectious diseases. Only recently, we have entered in a new phase of discovery of new vaccines but the scientific challenges are taxing our efforts to discover vaccines for many conditions. On the other hand, new excitement about several immunotherapeutic approaches to cancer is making a huge advance in our ability to combat those diseases. An exciting future is around the corner. Vaccine development and deployment, on the other hand still face many challenges and need equally aggressive approaches.

## Conflict of interest

None declared.
